# Mechanism of Body Weight Reducing Effect of Oral Boric Acid Intake

**DOI:** 10.1155/2013/914651

**Published:** 2013-06-03

**Authors:** Erhan Aysan, Fikrettin Sahin, Dilek Telci, Merve Erdem, Mahmut Muslumanoglu, Erkan Yardımcı, Huseyin Bektasoglu

**Affiliations:** ^1^Bezmialem Vakif University, ATA-2 Sitesi Akasya Caddesi No. 25 Cengelkoy, Uskudar, 80700 Istanbul, Turkey; ^2^Department of Genetics and Bioengineering, Yeditepe University, Turkey

## Abstract

*Objective*. The effect of oral boric acid intake on reducing body weight has been previously demonstrated although the mechanism has been unclear. This research study reveals the mechanism. *Subjects*. Twelve mice were used, in groups of six each in the control and study groups. For five days, control group mice drank standard tap water while during the same time period the study group mice drank tap water which contains 0.28 mg/250 mL boric acid. After a 5-day period, gene expression levels for uncoupling proteins (UCPs) in the white adipose tissue (WAT), brown adipose tissue (BAT), and skeletal muscle tissue (SMT) and total body weight changes were analyzed. *Results*. Real time PCR analysis revealed no significant change in UCP3 expressions, but UCP2 in WAT (*P*: 0.0317), BAT (*P*: 0.014), and SMT (*P*: 0.0159) and UCP1 in BAT (*P*: 0.026) were overexpressed in the boric acid group. In addition, mice in the boric acid group lost body weight (mean 28.1%) while mice in the control group experienced no weight loss but a slight weight gain (mean 0.09%, *P* < 0.001). *Conclusion*. Oral boric acid intake causes overexpression of thermogenic proteins in the adipose and skeletal muscle tissues. Increasing thermogenesis through UCP protein pathway results in the accelerated lipolysis and body weight loss.

## 1. Introduction

Boron, immediately to the left of carbon atom in the periodic table, is so similar to carbon that many carbon-based molecules are for all practical purposes the same as boron-based molecules. Boron is a stable metal found in nature as borate. The actual mean daily intake of boron in human diet is estimated to be 1.2 mg/day [1]. Boron is used in a wide range of products, including glass, detergents, fire retardants, and fibers to reinforce the plane fuselages and body armor, and in other superhard materials [2]. The similarity of boron to carbon has led to its wide use in biology [1, 3–5]. 

Recently we demonstrated that oral boric acid intake lowered the body weight of BALB/c outbred female mice [6]. Its mechanism, however, remained unclear. In this research we aim to reveal the molecular mechanism underlying the body weight reducing the effect of oral boric acid intake. To do so, fresh white adipose tissue (WAT), brown adipose tissue (BAT), and skeletal muscle tissue (SMT) were dissected from mice in the control group without the boric acid intake and the experimental group with the boric acid intake and we analyzed for the changes in the expression levels of uncoupling proteins (UCPs) 1, 2, and 3 using the real time PCR analysis.

UCPs are transmembrane proteins in the inner membranes of mitochondria and are responsible for the mitochondrial proton leak. Proton transport through UCPs lowers the mitochondrial membrane potential, leaving fewer protons to be transferred down the electrochemical gradient by the F0/F1 ATP synthase. 

The proton electrochemical gradient is dissipated as heat rather than being converted into ATP, resulting in the mobilization of fatty acid stocks without a consequent increase in ATP production [7]. Emerging evidence has suggested that differences in UCP expression among individuals may be responsible for the wide range of metabolic rate among populations. Reduced levels of UCP mRNA were associated with the increased risks for obesity in mice and humans [8–10]. 

In this paper, we demonstrate for the first time that the oral administration of boric acid induces an increase in the expression levels of UCP2 in WAT, BAT, and SMT cells also UCP1 in BAT cells. 

## 2. Methods

This study was performed in the Bezmialem Vakif University Experimental Animals Research Laboratory and Yeditepe University Genetics and Bioengineering Department Laboratories. Research protocol was approved by the Bezmialem Vakif University Local Animal Ethics Committee. All steps in this research were in accordance with the regulations governing the care and use of laboratory animals as set forth in the Declaration of Helsinki. 

Twelve outbred produced, 8-week-old female BALB/c mice were divided into two groups. According to power analysis with 0.05 accuracy and 0.95 power, the number of mice was determined as 6 each in both the control and study groups. The animals were kept in standard metabolic cages designed specifically for mice. A daily cycle of 12 hours of light/and 12 hours of dark was used for illumination of the room where mice were placed. 

For five days, mice were fed ad libitum with standard pellet feed manufactured specifically for small animals. The study group mice ingested boric acid through their drinking water: 0.28 mg boric acid (2 mg Bor Atac DF, %14 Boric Acid, TMT Co, Tekirdag, Turkey) was added to 250 mL tap water and dissolved by 3 minutes of agitation.

On days 0 through 5, all the mice were weighed every afternoon and the data were recorded. After five days, the animals were sacrificed by means of cervical dislocation.

WATs were isolated from omentum and SMTs were isolated from anterior abdominal muscles via anterior midline abdominal incision. BATs were isolated from interscapular area via posterior midline thoracocervical incision. 

Tissues were evaluated for changes in the expression levels of UCPs 1, 2, and 3 using the real time polymerase chain reaction (PCR) analysis. The primary evaluation parameter of this research was analysis of alterations of gene expression levels for UCP1, UCP2, and UCP3 in WAT, BAT and SMT cells. The secondary evaluation parameter was total body weight change observed in both groups of mice.

## 3. RNA Isolation and Real Time PCR Analysis

Total RNA from the fresh WAT, BAT, and SMT was isolated using peqGOLD RNAPure reagent (Peqlab, Germany) according to the manufacturer's instructions. Quality of RNA was checked by spectrophotometry and agarose gel electrophoresis. cDNA synthesis was performed using RevertAid First Strand cDNA Synthesis Kit (Fermentas, Lithuania) at 42°C for 60 min and 70°C for 5 min as described by the manufacturer. Real time PCR was performed with QuantiTect SYBR green PCR kit (Qiagen, Germany) according to instructions using 0.3 *μ*M primer concentration. PCR included 94°C for 15 min initial denaturation step followed by 40 cycles of 94°C for 30 sec, annealing for 1 min (61°C for UCP1 and 18S rRNA, 58°C for UCP2, 55°C for UCP3) and 72°C for 1 min, and a final extension step 72°C for 10 min. Each sample was analyzed in triplicate and the specificity of products was checked by the melt curve analysis. Results were analyzed by the standard curve method using the 18S rRNA house-keeping gene for normalization. The sequences of the primers were as follows: for 18S rRNA, sense 5′-AACTGAGGCCATGATTAAGAGG-3′, antisense 5′-GGCATCGTTTATGGTTGGAAC-3′; for UCP1 sense 5′-CTCGGGTCCTGGAACGTCAT-3′, antisense 5′-CAACGGAGCTGTTCATTTGATTTC-3′; for UCP2, sense 5′-GCTGGTGGTGGTCGGAGATA-3′, antisense 5′-ACAGTTGACAATGGCATTACGG-3′ [10] and for UCP3 sense 5′-GGCTGCCTGGAACAGAACAA-3′ and antisense 5′-TCCCATCAGGTCAGTGCAAAAC-3′. All real time PCR experiments were performed using iCycler iQ real time PCR detection system (Bio-Rad, USA).

## 4. Statistical Analysis

Statistical analyses were performed using SigmaStat Software. Results were evaluated with a confidence interval of 95% and a *P* < 0.05 level. In addition to descriptive statistical methods (mean, standard deviation, and median), the Mann-Whitney *U* test was used for the inter-group comparisons and the Wilcoxon test for comparison of in-group variables.

## 5. Results

**Figure 1:**
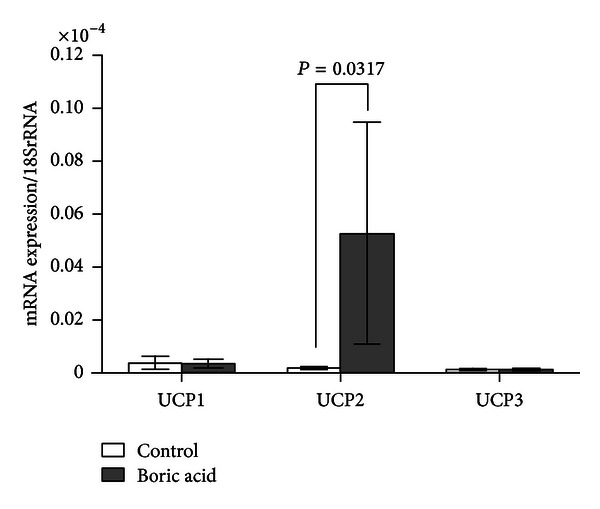
Real time PCR analyses in WAT (*P*: 0.0317).

**Figure 2:**
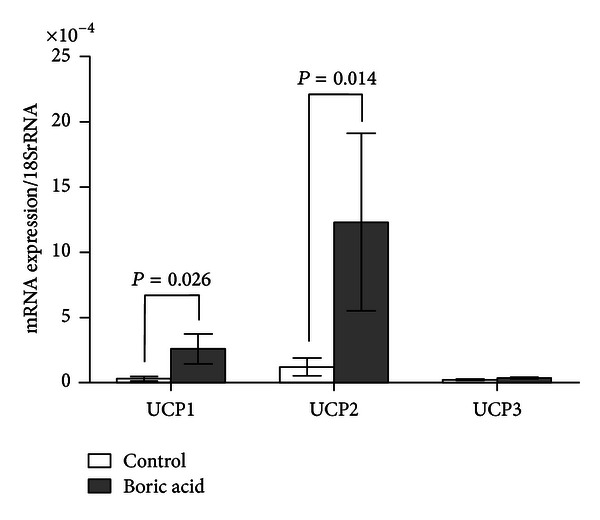
Real time PCR analyses in BAT (*P*: 0.014).

**Figure 3:**
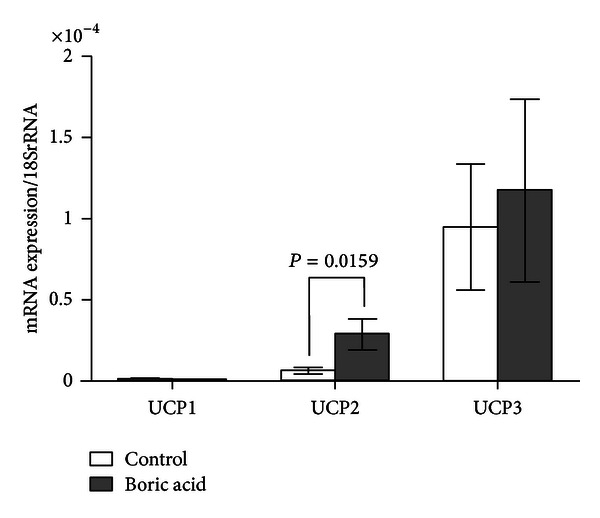
Real time PCR analyses in SMT (*P*: 0.0159).

Real time PCR analyses revealed that boric acid intake increased the UCP2 expression in WAT by 27-fold (*P*: 0.0317, Figure 1), BAT by 10.5-fold (*P*: 0.014, Figure 2), and SMT by 4.5-fold (*P*: 0.0159, Figure 3) (Table 1). In addition, boric acid intake led to a 9-fold (*P*: 0.026) increase in UCP1 expression in BAT (Figure 2). UCP1 in the other two tissues and UCP3 in all three tissues were not overexpressed significantly (*P* > 0.05, Figure 1, Table 1).

Body weight changes of the groups and statistical analyses were revealed in Table 2. Mice which were fed with boric acid lost their body weight at a mean of 28.1% in five days. In the control group, no weight loss was observed; indeed, a weight gain averaged at 0.09% was recorded. Body weight changes between the groups were statistically significant (*P* < 0.001). From day 0 through day 5, total body weight differences were statistically significant in the boric acid group (*P* < 0.005), but not in the control group.

## 6. Discussion 

It is clear that obesity is characterized by an imbalance between energy intake and expenditure. Emerging evidence has placed adipocytes at the center stage since alterations in the regulation of lipogenesis and lipolysis have been associated with obesity. Oberkofler et al. demonstrated that impaired adipose tissue expression of UCP2 may play a role in the pathophysiology of obesity [10].

UCP1 is a key protein in thermogenesis and regulation of energy expenditure mechanisms which are important in obesity [11]. Molecular players that are responsible for the control of UCP expression in adipocytes are still a subject of debate; however, hormonal stimuli such as glucagon and triiodothyronine treatment or feeding/fasting conditions have been demonstrated to affect the UCP-2 expression in adipocytes [7]. 

In our first-step research, as discussed above, we had revealed the effect of oral boric acid intake on reducing body weight [6]. In our previous research, we revealed that blood cholesterol, LDL, AST, ALT, LDH, amylase, and urine urobilinogen levels were statistically high in the boric acid intake group, and therefore we hypothesized that these results may be related to an increased catabolism with especially high lipid consumption. Now in the second step of our research, we have revealed the mechanism: the boric acid intake led to the over expression of UCPs (UCP1 in BAT, UCP2 in WAT, BAT, and SMT) resulting in an increase in thermogenesis and an acceleration of lipolysis.

The function of adipocytes is the storage of triacylglycerols as energy reserve in the body and maintenance of the systemic energy balance through the regulation of lipogenesis and lipolysis. In addition, adipocytes such as the ones found in the brown fat tissue are involved in the thermoregulatory thermogenesis where the energy from the cellular respiration is transferred into heat by the action of the mitochondrial uncoupling proteins (UCP-1). UCP-1, the first UCP isolated and identified, is highly expressed in the brown fat tissue [12] and involved in uncoupling of proton transport from ATP synthesis hence increase the demand for fuel substrates such as glucose and lipids in the oxidative phosphorylation processes [13]. Additions to the UCP family came with the discovery of the homologues UCP2 and UCP3 [14, 15]. While UCP2 was found to be widely expressed in different range of cells, UCP3 expression was mainly restricted to skeletal and cardiac muscle and brown fat [7]. Although the contribution of UCP2 and UCP3 in nonshivering thermogenesis is debatable due to their low expression ratios in the membrane, recent reports suggested that UCP2 and UCP3, similar to UCP1, can lower the proton electrochemical gradient across the mitochondrial membrane causing a decrease in ATP production when overexpressed in mammalian cells [13, 16].

Although effects of boron on metabolism and enzymes were known, its body weight reducing effect through UCP1 and UCP2 overexpression was not demonstrated before. It is clear that obesity is characterized by an imbalance between energy intake and expenditure. Emerging evidence has placed adipocytes at center stage since alterations in the regulation of lipogenesis and lipolysis have been associated with obesity. For example, isolated adipocytes from obese patients showed a twofold decrease in the specific activity of the lipogenic marker enzyme Glyceraldehyde-3-Phosphate Dehydrogenase (G3PDH) when compared to that of nonobese patients, resulting in a lower basal lipolytic rate [17]. There is also recent evidence relating the reduced UPC2 expression to morbid obesity hence increasing the emphasis on the importance of adipocytes in maintaining homeostasis of energy dissipation and storage [9]. Molecular players that are responsible for the control of UCP expression in adipocytes are still a subject of debate. However, hormonal stimuli such as glucagon and triiodothyronine treatment or feeding/fasting conditions have been demonstrated to affect the UCP-2 expression in adipocytes [7]. 

In conclusion our results are promising and may open a new line inquiry for the boron-based prevention or treatment approaches to obesity. Some questions await answers, such as complications of long-term boric acid intake. The effect of boric acid intake must also be evaluated as to different means (local, muscular, or intravenous) and with different doses. As UCPs may not be as active in other animals such as rat and rabbit as it was in mice, their effect should also be tested in these model animals. Taken together, boric acid intake may become a new and effective way to treat the worldwide health problem of obesity.

## Figures and Tables

**Table 1:** The mean gene expression levels normalized against 18sRNA ± SE expression for each UCP proteins in WAT, BAT, and SMT.

	Boric acid group	Control group	*P* value
UCP1 mRNA in WAT	0.00000017 ± 0.00000004	0.00000015 ± 0.00000008	0.6905
UCP2 mRNA in WAT	0.00000527 ± 0.00000420	0.00000019 ± 0.00000006	0.0317
UCP3 mRNA in WAT	0.00000013 ± 0.00000007	0.00000012 ± 0.00000006	0.9452
UCP1 mRNA in BAT	0.00025809 ± 0.00011527	0.00002859 ± 0.00001483	0.026
UCP2 mRNA in BAT	0.00123186 ± 0.00067987	0.00011691 ± 0.00006580	0.014
UCP3 mRNA in BAT	0.00002918 ± 0.00000762	0.00001676 ± 0.00000534	0.2949
UCP1 mRNA in SMT	0.00000071 ± 0.00000022	0.00000084 ± 0.00000063	0.366
UCP2 mRNA in SMT	0.00002884 ± 0.00000967	0.00000645 ± 0.00000198	0.0159
UCP3 mRNA in SMT	0.00011747 ± 0.00005621	0.00009499 ± 0.00003876	0.7308

**Table 2:** Body weight changes of the groups and statistical analyses in five days.

Groups	Day 0	Day 5	Difference	Wilcoxon Test	
				*z*	*P *
Control Group					
Mean	21.50	21.92	0.42	−1.890	0.059
Std. Deviation	1.05	1.32	0.38		

Boric Acid Group					
Mean	21.83	15.92	−5.92	−2.220	0.026
Std. Deviation	1.17	0.80	0.66

Mann-Whitney Test			
*z*	−.580	−2.892	−2.929		
*P*	.562	.004	.003		
